# TreeTerminus —creating transcript trees using inferential replicate counts

**DOI:** 10.1016/j.isci.2023.106961

**Published:** 2023-05-25

**Authors:** Noor Pratap Singh, Michael I. Love, Rob Patro

**Affiliations:** 1 Department of Computer Science, University of Maryland, College Park, MD, USA; 2 Department of Biostatistics, University of North Carolina, Chapel Hill, NC, USA; 3 Department of Genetics, University of North Carolina, Chapel Hill, NC, USA

**Keywords:** Bioinformatics, Data processing in systems biology, Transcriptomics

## Abstract

A certain degree of uncertainty is always associated with the transcript abundance estimates. The uncertainty may make many downstream analyses, such as differential testing, difficult for certain transcripts. Conversely, gene-level analysis, though less ambiguous, is often too coarse-grained. We introduce TreeTerminus, a data-driven approach for grouping transcripts into a tree structure where leaves represent individual transcripts and internal nodes represent an aggregation of a transcript set. TreeTerminus constructs trees such that, on average, the inferential uncertainty decreases as we ascend the tree topology. The tree provides the flexibility to analyze data at nodes that are at different levels of resolution in the tree and can be tuned depending on the analysis of interest. We evaluated TreeTerminus on two simulated and two experimental datasets and observed an improved performance compared to transcripts (leaves) and other methods under several different metrics.

## Highlights

• TreeTerminus outputs transcript-trees for an RNA-Seq experiment in data-driven manner
• Uncertainty decreases on average across the experiment, ascending the tree topology
• Specific objective functions can be optimized on the tree to obtain discrete groups
• Flexibility to analyze data at different levels of resolution

## Graphical abstract

**Figure:**
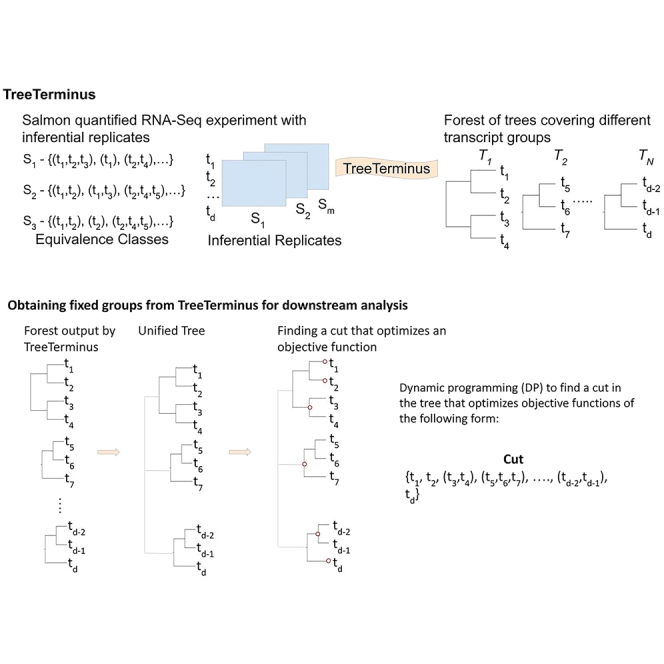

## Introduction

Transcript abundance estimation is among the key target applications of RNA-Seq. A gene can express multiple transcripts due to alternative splicing where a combination of exons and introns can be joined in a different manner. The variation in transcript expression plays a key role in development^1^^,^^2^^,^^3^; and characterization of diseases and their subtypes.^4^^,^^5^^,^^6^ One approach to estimating transcript abundances is to probabilistically assign a given fragment to the transcripts using maximum likelihood or Bayesian inference.^7^^,^^8^^,^^9^ This has to be done since there exists an ambiguity toward finding the true locus of origin for a given sequencing fragment when it can map equally well to the shared sequences within transcripts. Thus, a certain degree of uncertainty is associated with the point estimates of transcript abundance, depending on the nature of the fragments. This in turn makes downstream analysis, such as differential testing, difficult for certain transcripts and impacts their accuracy. Uncertainty also exists for gene expression estimates since the sequencing fragments can come from the shared sequences within genes, however, gene expression estimates will be less ambiguous than transcripts. The uncertainty of a transcript/gene can be estimated from the inferential replicates generated either through MCMC/Gibbs Sampling; or through a bootstrap sampling of the reads and rerunning the abundance estimation algorithm for each bootstrap replicate.^7^^,^^8^^,^^9^^,^^10^^,^^11^

The highly uncertain transcripts/genes may become invisible when the inferential replicates are incorporated into the downstream tasks. To circumvent this problem, some methods have grouped transcripts/genes into distinct inferential units that share a lot of multi-mapping reads.^12^ mmcollpase^13^ proposes to group transcripts by computing the correlation between every pair of transcripts on the posterior replicates and the pair that has the most negative correlation is grouped. This process is repeated for multiple iterations until a stopping criterion is reached, where at each iteration the correlation is recomputed for every transcript/group with the group from the previous iteration and a new group is formed. Terminus^14^ also provides transcript groups as an output but employs difference in inferential relative variance^15^ between the group and the mean of its underlying transcripts/subgroups as the statistic for group creation. It first creates a graph on the transcripts, with an edge denoting that the transcripts co-occur in at least one range-factorized equivalence class^16^ and only the transcripts that are connected by a path on the graph are considered for grouping. Terminus first finds groups across each individual sample and uses a consensus approach to output consensus groups across samples. Notably, both mmcollapse and Terminus provide a single level of resolution for analysis, not permitting analysis across different levels.

Another limitation with both of the above methods is that the resolution at which grouping is stopped is governed by a threshold determined heuristically that does not take the downstream analysis into account. When performing downstream tasks such as differential analysis, these methods can either thus over-aggregate transcripts, masking the signal in the process or under-aggregate leading to weaker signals, and an inability to make confident calls. Since the goal of these methods is to provide concrete groups as output without doing much aggregation, they introduce many filtering constraints on transcripts in order for them to be considered for aggregation which might further lead to missing good candidates.

In this work, we introduce TreeTerminus, a new method that aims to address some of the shortcomings mentioned previously. TreeTerminus expands upon the idea of Terminus and groups transcripts in a tree structure where leaves represent individual transcripts and internal nodes represent an aggregated set of leaf transcripts. Across the samples in the experiment, inferential uncertainty decreases on average, ascending the tree topology. TreeTerminus can be run on a single sample and we provide two different approaches in order to extend it for the multi-sample settings. The tree provides the flexibility to analyze data at nodes that are at different levels of resolution in the tree and can be tuned depending on the analysis of interest. Thus, TreeTerminus provides the ability to represent the structure of the inferential uncertainty present in the data, and to convey this structure to methods for downstream analysis. While this representation of RNA-seq quantification data are quite new, we believe that it will be important in helping to develop more accurate approaches for problems like differential testing, where testing procedures may eventually choose to convey results at a level that maximizes resolution while simultaneously reducing the inherent quantification uncertainty. The groups of transcripts tested can be linked by biologically meaningful characteristics, like sharing a particular sequence of exons or a common transcription start site, though these annotations need not be known or provided as input to the method. To obtain fixed groups for downstream analysis, we provide a dynamic programming (DP) approach that can be used to find a cut through the tree that optimizes one of several different objectives. In addition, the DP approach has the ability to optimize for other user-defined objective functions provided they adhere to the required constraints necessary to be efficiently optimized on a tree. The inner nodes that are obtained from solving the DP might contain those transcripts which would not have been recoverable by doing the analysis at the transcript level, but whose signal can be preserved at a higher level that is provided by the inner nodes.

To the best of our knowledge, this is the first time that transcripts have been arranged in a tree-like structure, that too in a data-driven manner. We evaluated TreeTerminus on two simulated and two experimental datasets and observed an improved performance compared to transcripts (leaves) and other methods under several different metrics. TreeTerminus has been implemented in Rust. We have also created an R Package beaveR that parses the output of TreeTerminus, implements DP algorithms for finding an optimal cut, and provides helper functions to obtain useful statistics for subtrees within the TreeTerminus-derived tree structures.

## Results

### Overview of TreeTerminus

**Figure 1:**
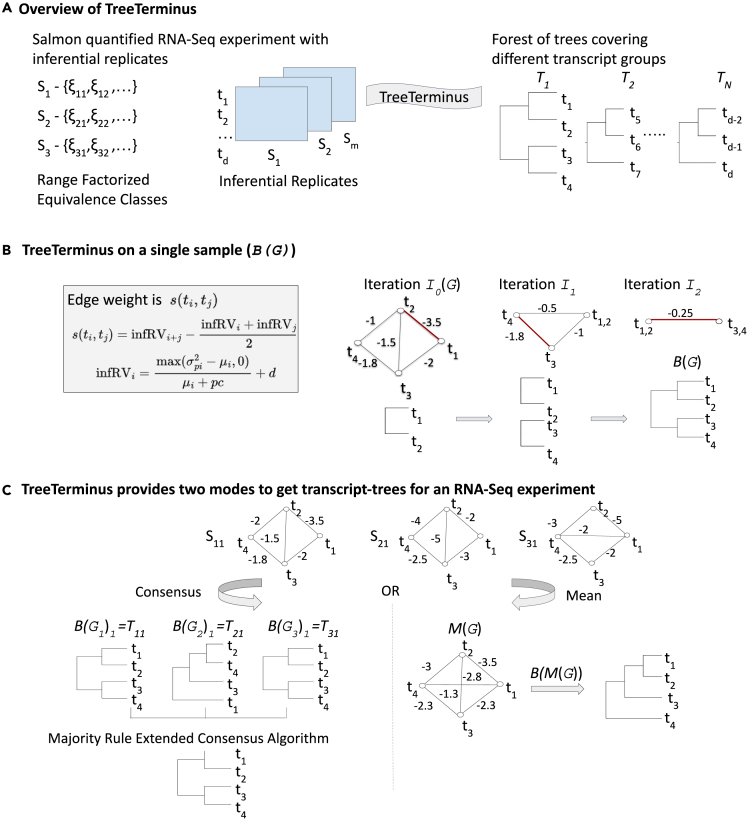
Schematic overview of TreeTerminus pipeline (A) Taking an RNA-Seq experiment as an input, that has been quantified with Salmon and for which inferential replicates have been generated, TreeTerminus outputs a forest of trees. (B) Toy example demonstrating how from the transcript graph (*G*), how a forest shall be generated by TreeTerminus for a single sample using the procedure B(G). A forest will consist of multiple trees when the graph consists of disconnected components or some of the necessary conditions that are required for aggregating transcripts/groups are not met. The color red indicates the edge with the lowest weight chosen for aggregation at each iteration. (C) When there are multiple samples—TreeTerminus provides two modes to generate the forest, Mean and Consensus. In the toy example, both procedures are demonstrated using a single transcript group, thus the forest consists of a single tree.

We briefly describe TreeTerminus pipeline as shown in Figure 1. Using the abundance estimates and inferential replicates for an RNA-Seq experiment that are generated by Salmon, TreeTerminus outputs a forest of trees, where leaves represent individual transcripts and internal nodes represent an aggregation of a transcript set. The trees are constructed such that, on average, the inferential uncertainty decreases as we ascend the tree branches. To construct trees for a single sample, first, a graph is created on the set of transcripts, with an edge denoting that they co-occur in at least one range-factorized equivalence class for that sample. The edge weight denotes the reduction in inferential uncertainty (Equation 1 in STAR Methods) that will be observed if the two transcripts/transcript groups were to be merged. The edges are then greedily collapsed from the graph creating a forest of trees, each representing a transcript group. The group consist of transcripts, such that between any two transcripts there exists a path between them in the initial transcript graph at iteration $I_{0}$. TreeTerminus provides two modes to output a forest of transcript trees across the set of samples for an RNA-Seq experiment. The Mean tree uses the mean reduction in inferential variance across samples as the edge weight for aggregating nodes, whereas the Cons tree applies a majority rule extended consensus algorithm on the individual trees obtained across samples for a given transcript group.

### Experimental setup

#### Datasets

We ran TreeTerminus on two simulated and two experimental bulk RNA-Seq datasets.^17^^,^^18^ TPM (Transcripts per Million) estimates extracted from GTEx V8 frontal cortex dataset were used as an input to generate the simulated datasets, containing 12 samples in 2 conditions with 6 samples in each. Two simulated data variations were created and referred to as BrSimNorm and BrSimLow, respectively. The first experimental dataset is derived from two different mouse muscle tissues, consisting of 6 samples in each and we refer to it as MouseMuscle. The second experimental dataset is obtained for the brain tissues of Chimpanzee, consisting of a total of 73 samples. The two groups for our analysis consist of 5 and 68 tissues, respectively. The resource usage and time performance comparing Terminus to TreeTerminus are provided in Tables 1 and S2.

**Table 1:** Peak memory usage and running time for the different steps of Terminus and TreeTerminus (both Mean and Cons modes) on BrSimNorm dataset

Method	Terminus		TreeTerminus (Mean)	TreeTerminus (Cons)	
	Group	Consensus	Group	Group	Consensus
Peak Memory (MB)	234	2721	3340	435	458
Time (h:m:s)	0:23:40	0:02:29	0:03:08	0:20:14	0:17:26

#### Creation of a baseline anti-correlation tree

For comparison with TreeTerminus, we are unaware of any other existing methods that create transcript-trees. Thus, to create a baseline tree, we create a tree using Anti-Correlation (AC) (negative correlation) between transcripts computed on the inferential replicates, which we also refer to as the AC tree. The motivation for using AC comes from mmcollapse,^13^ which was also used as a baseline method for comparison in Terminus.^14^ Since mmcollapse does not create trees, we use UPGMA.^19^ UPGMA applies a hierarchical clustering procedure on the leaf set to generate a tree, which based on the current input, will group an anti-correlated set of transcripts, leading to a reduced uncertainty. We thus believe the UPGMA tree is a fair baseline for comparison with the TreeTerminus trees where uncertainty decreases as we ascend the tree.

#### Different tree methods that have been compared in this study

In this study, we have used both the variations of TreeTerminus, where a unified tree is constructed from the forest of Cons and Mean trees. We evaluate their performance on several parameters. To see if there are any benefits of removing the constraints imposed in Terminus, we also compare with the unified trees obtained after running the consensus algorithm on the sample trees that were constructed with the variations of those constraints (see STAR Methods). In the first variation, the consensus tree approach is applied on trees obtained using both constraints i.e. Filt and ES (Early Stop) which is referred to by ConsFiltES. In the second variation, we only apply the constraint Filt and the corresponding consensus tree that is created is referred to by ConsFilt. The AC tree serves as the baseline tree. We create trees for all the aforementioned methods for both the simulated and experimental datasets. We also compare with Terminus groups (Term) and transcripts (Txp) whenever possible. The total number of transcripts covered by different methods across the different datasets is provided in Table 2. Mean tree covers the most transcripts followed closely by Cons tree while ConsFiltES tree and Term cover the least number of transcripts. To facilitate the comparison between the methods, we ensure that all trees cover the same transcripts. A transcript set is created by taking the union of the transcripts covered by the trees in the set {Mean,Cons,ConsFilt,ConsFiltES,AC}, and groups in Term, with Txp referring to that transcript set. To the children of the root node of the above trees, we append any transcripts that were present in Txp but missing in the transcripts covered by the individual trees. Similarly, nodes in Term consist of all nodes output by Terminus along with any transcripts present in Txp but not covered by Terminus groups.

**Table 2:** Total number of transcripts covered by different methods across the datasets

Method	BrSimNorm	BrSimLow	MouseMuscle	ChimpBrain
Mean	135138	135208	98091	39856
Cons	134377	134371	97360	39491
ConsFilt	121784	121657	89429	37858
AC	73205	73493	68062	35114
ConsFiltES	27156	26891	17939	15773
Term	13612	13730	7792	5428

### TreeTerminus nodes have the lowest mean inferential variance

**Figure 2:**
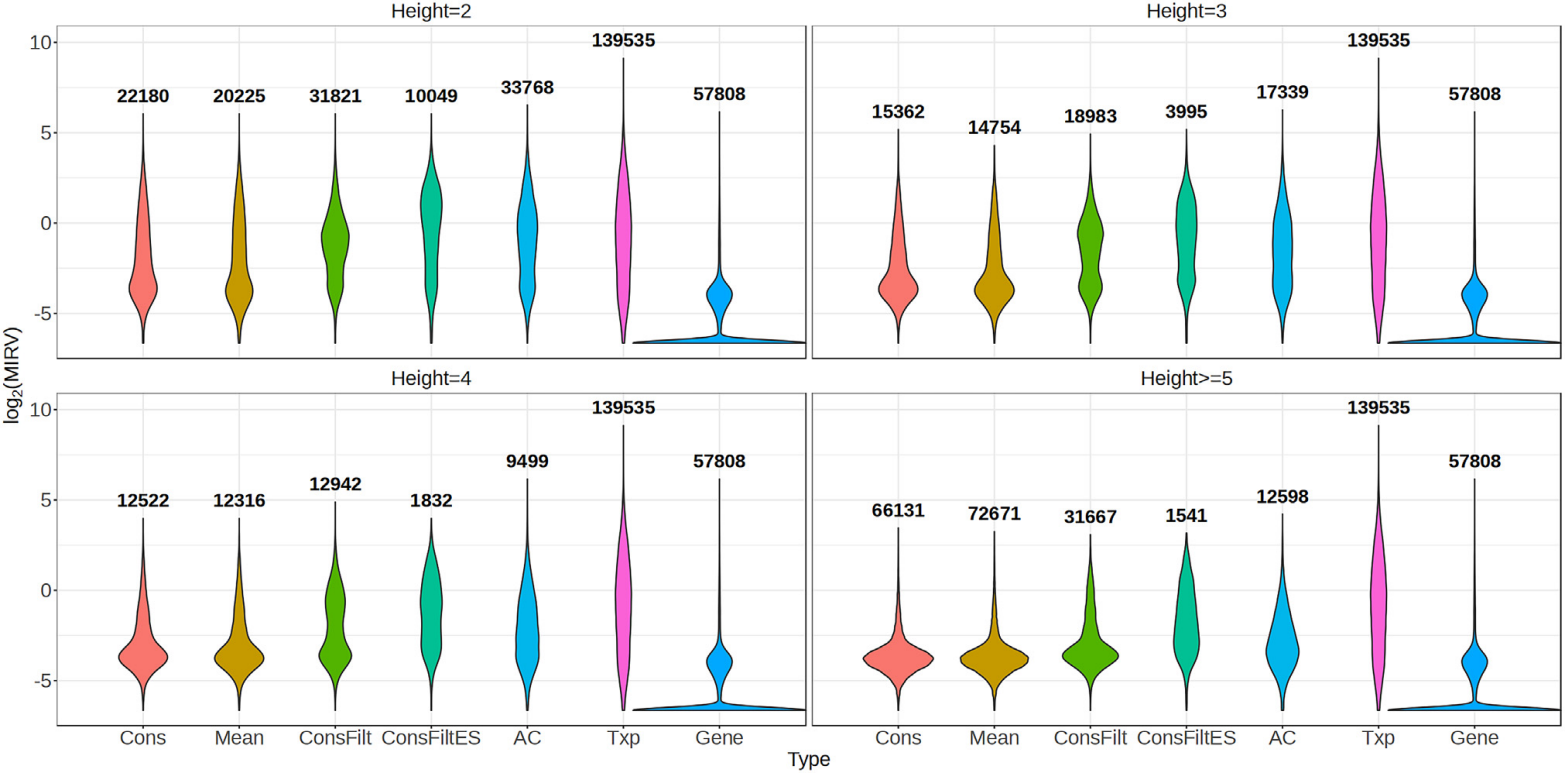
Distribution of $\log_{2}$ MIRV(mean inferential relative variance) across the inner nodes for the BrSimNorm dataset, stratified by their height The total number of inner nodes belonging to a method at a given height is written on top of the violin plot. Also plotted for comparison at each height is the distribution of *lg* of MIRV for the transcripts and genes.

The violin plot in Figure 2 compares the distribution of $\log_{2}$ MIRV (mean inferential relative variance) for the inner nodes across trees stratified by their height for the BrSimNorm dataset. For comparison, $\log_{2}$ MIRV for genes and transcripts has been plotted along with the trees at each height. The transcripts have the highest range of variation in the MIRV and as expected have the largest values. Genes have the lowest MIRV with the majority of genes having the lowest possible value of 0.01 that can be obtained using the default thresholds (Equation 2). Among the trees, the nodes of Cons and Mean trees have the lowest MIRV across different heights followed by ConsFilt and AC trees. The nodes belonging to ConsFiltES tree have the highest MIRV. As expected, going up the tree, MIRV decreases across the methods getting closer to gene MIRV levels. These trends are summarized by the median values of MIRV in Table S3. The previous pattern is also repeated in the BrSimLow dataset(Table S4 and Figure S3). A similar trend is observed for the MouseMuscle dataset (Figure S4 and Table S5). The only change is observed on the nodes at height greater than or equal to 5 for the ConsFiltES tree which shows the most downward shift in MIRV distribution. It is important to note that across all the datasets, ConsFiltES has the lowest number of nodes. For the ChimpBrain dataset, a very similar distribution is observed for the trees across all the methods and a modest downward shift in the upper tail is observed as the height increases. The MIRV though is very low for the height 2 inner nodes to begin with (Table S6 and Figure S5). However, a considerable downward shift in the distribution of MIRV is still observed for the inner nodes compared to the transcripts.

### TreeTerminus nodes map to relatively fewer genes and gene families

**Figure 3:**
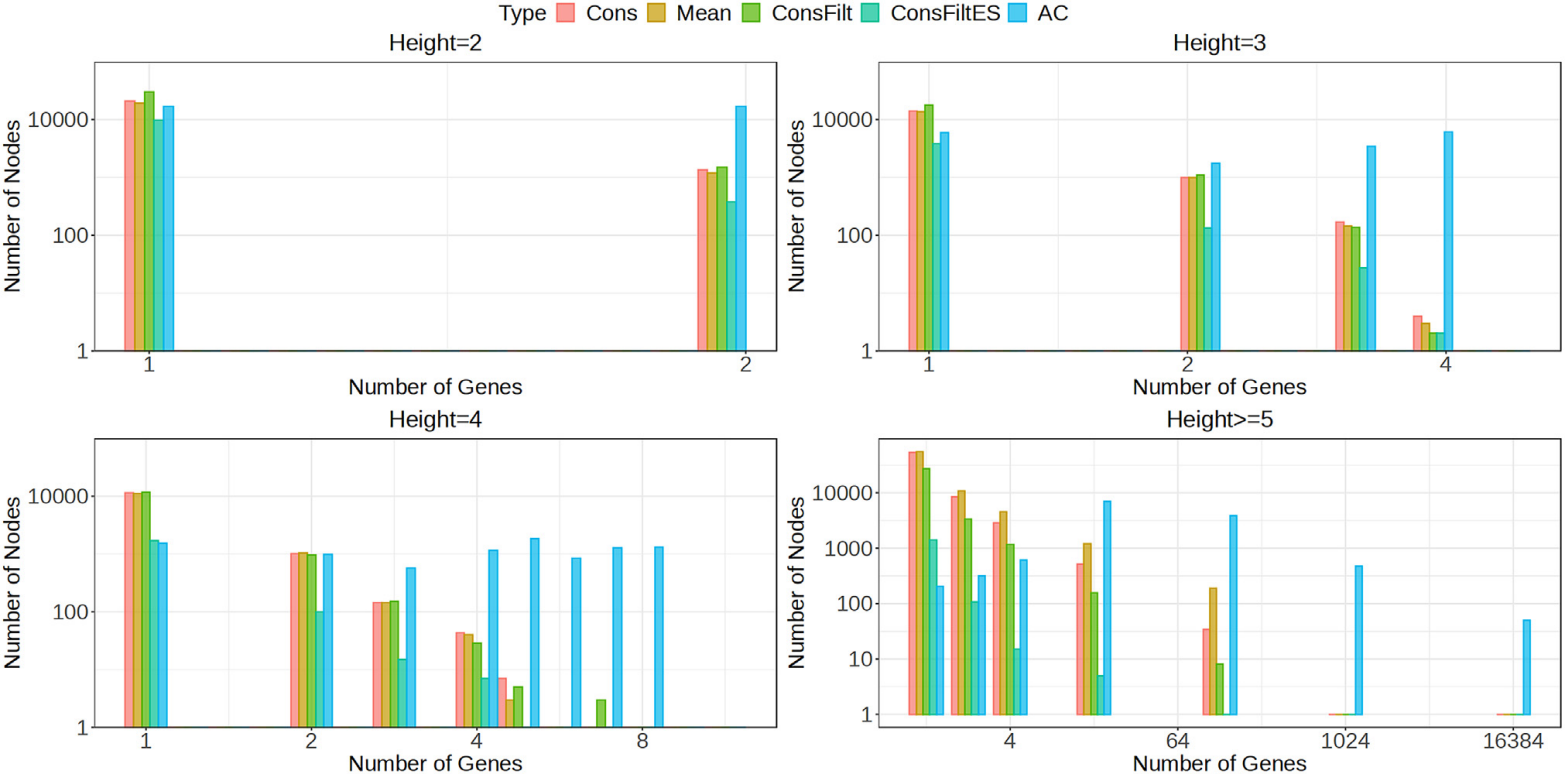
Comparison of different methods with respect to the number of genes to which an inner node in the tree maps for the BrSimNorm dataset, stratified by their height The x axis represents the number of unique genes that transcripts belonging to the inner nodes map to and the y axis represents the frequency of such mappings at a given height for a tree. For all the inner nodes located at a height greater than or equal to 5, the number of unique genes was binned using the set {1,2,4,16,128,1024,16384}, with the bin representing the number of unique genes less than or equal to the bin but larger than the bin left to it.

We next look at the distribution of the number of uniquely mapped genes at the inner nodes located at different heights across the trees. The mapped genes associated with a node are found by mapping the descendant leaves (transcripts) for that node to the genes. Figures 3, S6, S7, and S8 plots this distribution for the different datasets. The proportion of nodes that map to more than one gene is considerably higher for the AC tree compared to others, with the number of nodes at a height two that map to two genes, 10 to 100 of times larger for the AC trees. The increase in the relative number of multi-gene mapping nodes is also the highest for the AC tree and it has the highest magnitude of the number of unique genes to which an inner node can map. With the exception of the AC tree, the proportion of nodes that map to only one unique gene dominates nodes mapping to multiple genes across different heights in the other trees. The AC tree has the highest number of inner nodes that map to more than one hundred genes across the datasets (Table S7). No such nodes exist in the other trees obtained across the datasets, barring the MouseMuscle dataset, for which there exist a reasonable number of such inner nodes, with some even mapping to more than a thousand genes. When we looked at the genes mapping to these nodes, we found that a large proportion of such genes were either predicted or pseudogenes, which was not necessarily the case for most of such nodes in the AC tree.

**Figure 4:**
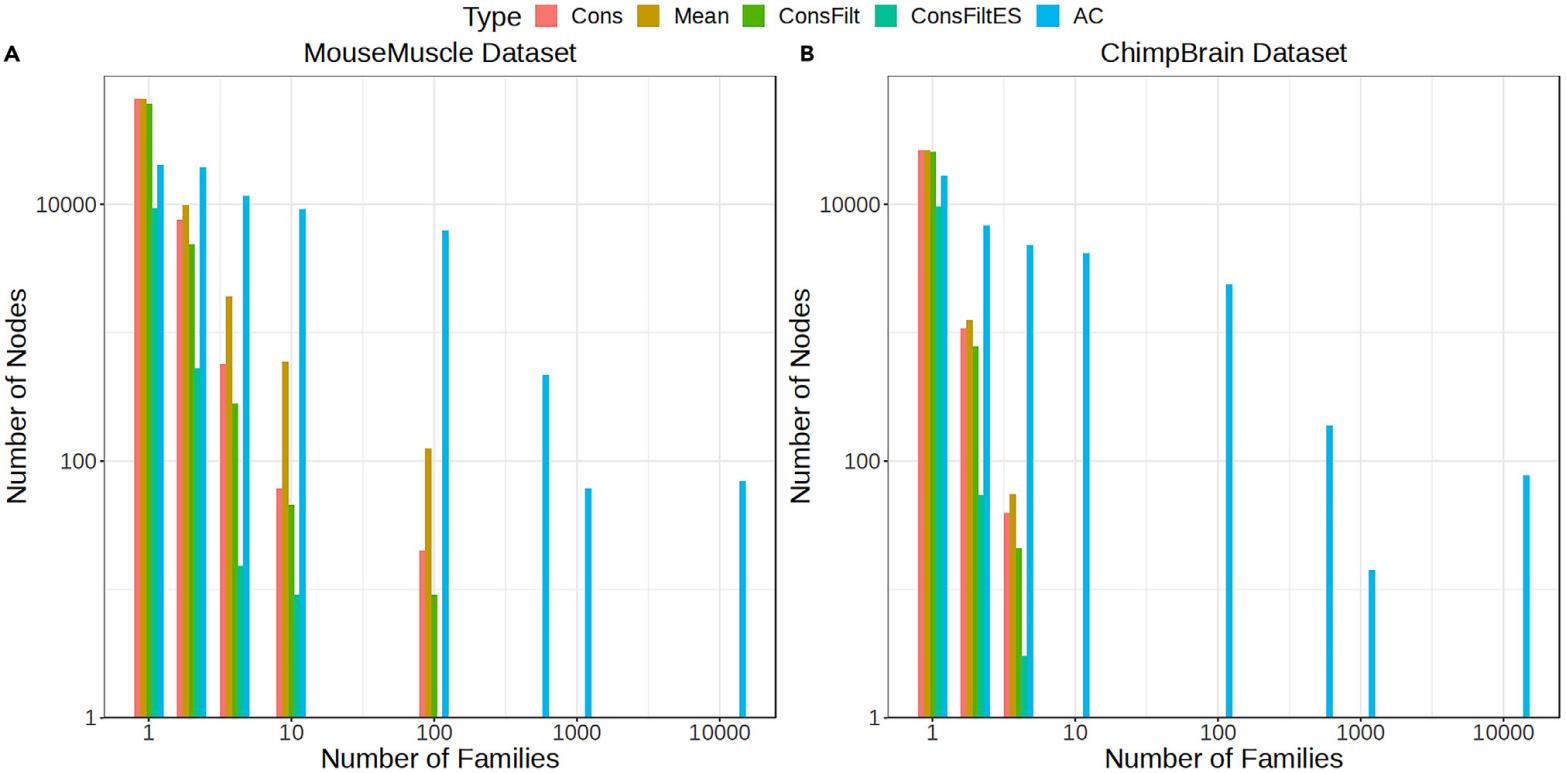
Comparison of different tree methods with respect to the number of gene families to which an inner node maps The number of gene families that map to an inner node has been binned into the set {1,2,4,10,100,500,1000,12000}, with the bin representing the number of unique gene families less than or equal to the bin but larger than the bin left to it. The x axis represents the bin and the y axis represents the total frequency of inner nodes mapping to the gene families in that bin for a given tree. This has been plotted for MouseMuscle and ChimpBrain datasets.

We also explored the distribution of the number of gene families to which inner nodes belonging to different trees map in Figure 4. The number of gene families have been binned into bin numbers {1,2,4,10,100,500,1000,12000} so that each bin represents the number of unique gene families less than or equal to the bin but larger than the bin left to it. For the MouseMuscle dataset, the number of nodes mapping to more than one family is the largest for the AC tree and the difference in the number of nodes increases between AC and the remaining trees as the magnitude of the number of gene families they map to is increased on the x axis. While the AC tree has more than 5000 nodes that map to (10,100] gene families, this number is only 124 for the Mean tree and much less for the other trees. There are more than 500 nodes that map to more than 100 gene families for the AC tree while hardly any such node exists for the other trees. A similar trend is observed for the ChimpBrain dataset, while for the AC tree there are more than 2500 nodes that map to more than 10 gene families, such nodes don’t exist for the other trees. The nodes belonging to the AC tree thus map to many more gene families. While the nodes belonging to the other trees map to fewer gene families, we have not explored them in the current work.

### Cuts obtained from TreeTerminus trees are among the top performers when optimizing for the different objective functions

**Figure 5:**
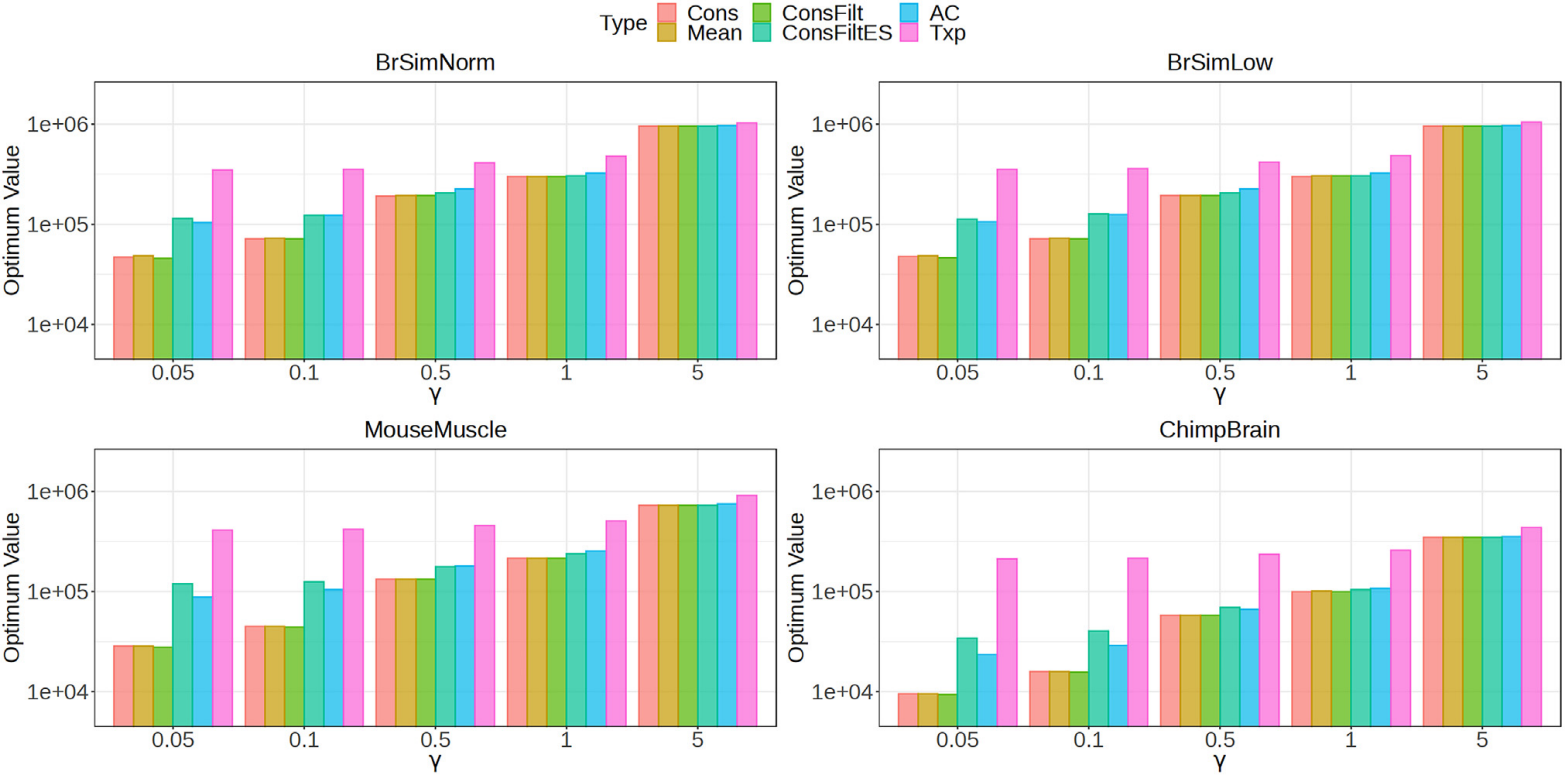
Barplot representing the values for the objective function on the cut obtained by minimizing the sum of metric—the sum of the metric (irv_height_desc) on the different datasets For each dataset, optimal values of the objective function are compared across the methods for a range of γ values. Also plotted for comparison is the optimal value obtained using transcripts (Txp) as the cut.

In order to do any downstream analysis and interpret its results, we require discrete inferential units. The discrete inferential units can be obtained by finding a cut on the tree that solves for an objective function which optimizes a node metric. The inferential unit that represents a transcript group might represent a stronger signal w.r.t a node metric that the end user is interested in optimizing as compared to aggregation for that metric on the individual transcripts contained in that group. The type of objective function that we optimize is described in detail in STAR Methods and requires a metric as input for every node in the tree. Specifically, in this manuscript, we have used two metrics to demonstrate the benefits of our proposed tree structure. For the first objective function, we want to find nodes that have a low MIRV and at the same time are at a level close to the leaf in order to provide the finest resolution for downstream analysis. We refer to this objective function as irv_height_desc (see STAR Methods). Figures 5 and S9 plots the optimal values and the cut sizes obtained by solving for the previous objective function on the different trees for each dataset. Also plotted for comparison are these values when the cut consists of only the transcripts(Txp).

The cuts for different trees have been obtained by using γ values from the set {0.05,0.1,0.5,1,5}. Increasing γ will provide more weight to the height, leading to cuts with lower height nodes. The highest (worst) optimal value is seen for the cut consisting of transcripts, whereas the cuts obtained for Cons, ConsFilt, Mean trees consistently have the lowest(best) optimal values. The relative difference in the optimal value between them is smaller compared to differences in optimal values for the cuts obtained from the other trees. As γ increases, the size of the cut increases as expected along with the increase in the optimal value. Further, the differences in the optimal values and cut sizes between the methods also decrease, with these values individually becoming comparable at γ=5 across the trees. We also looked at the distribution of the values of the metric in the cuts for the different trees and the transcripts in Figure S10. A large proportion of nodes for the Cons, Mean, ConsFilt trees show a very low value for the metric compared to the other methods, showing that the net value of the objective function is not dominated by just a few outlier nodes.

**Figure 6:**
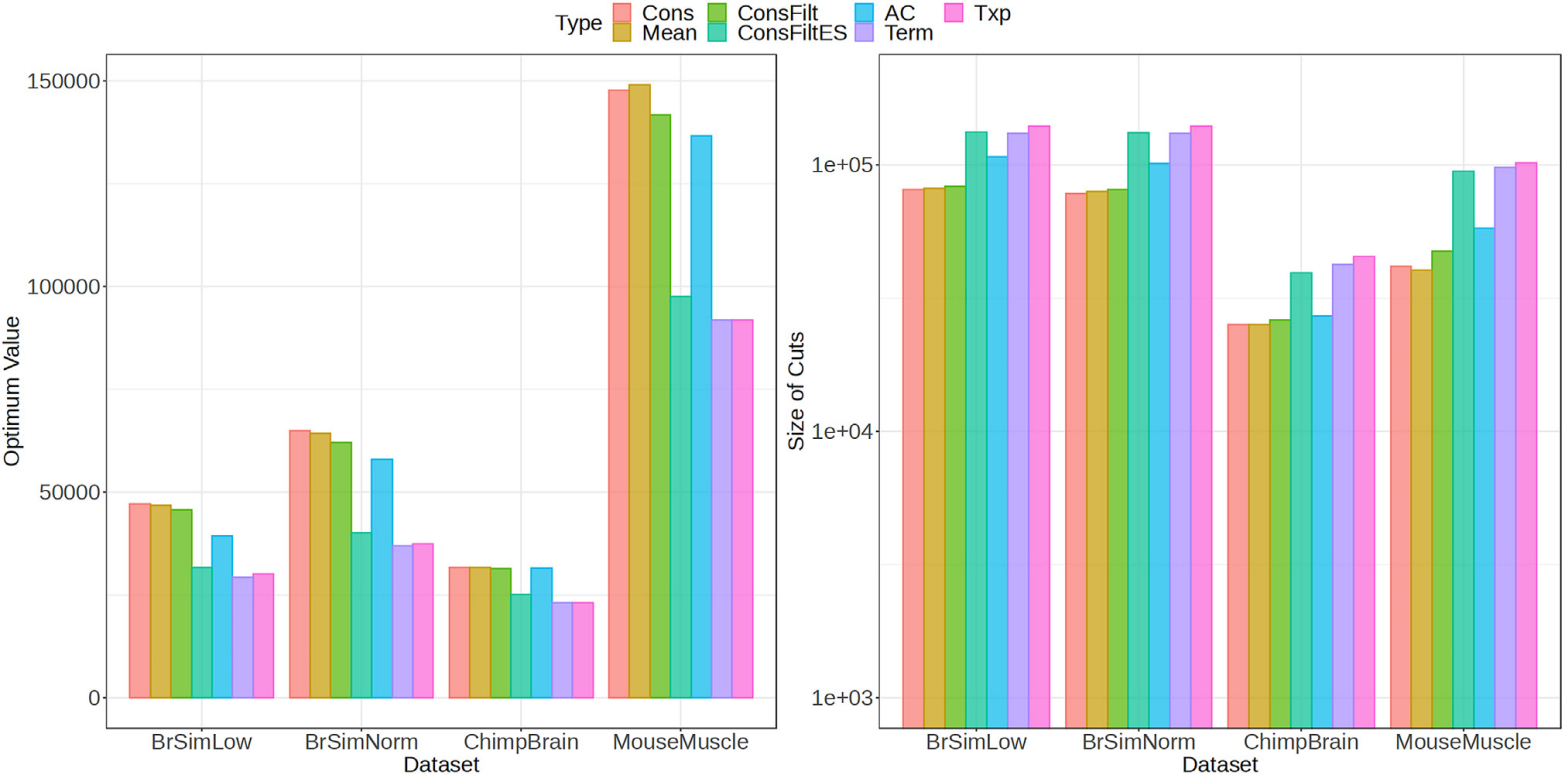
Comparing the performance on the cuts obtained by solving the objective function that maximizes the sum of metric (lfc_desc) for the nodes in a cut on the different datasets The performance is also compared when the transcripts and Term groups are taken as the cut. (A) Value of the objective function. (B) Size of the cut for the different trees.

For the second objective function, we find nodes that have a high log fold change and low MIRV. We refer to this objective function as lfc_desc(see STAR Methods) and plot the optimal values and cut sizes obtained for this objective for the different trees on each dataset in Figure 6. Also compared are the values that are obtained when the cut consists of—Terminus (Term) groups and the transcripts(Txp). The cuts obtained from the Cons tree have the highest (best) optimal value followed closely by the Mean tree. These are then followed by the cuts obtained for the ConsFilt and AC tree. The cuts obtained for ConsFiltES tree, Term and Txp have the lowest (worst) optimal values. An opposite trend is observed for the cut sizes, with Txp having the largest size followed by cuts from Term and ConsFiltES tree.

### An optimal performance is observed for differential expression analysis on the cuts obtained from TreeTerminus trees

We also evaluate the performance of doing differential expression analysis on the base inferential units consisting of cuts obtained from the Cons tree along with genes, transcripts and Terminus groups for the BrSimLow and BrSimNorm datasets (Tables 3 and S8). For the BrSimLow dataset, we observe that the genes have the lowest FDR (False Discovery Rate) and a high TPR (True Positive Rate), while the lfc_desc cut has the highest TPR. Similarly, the cuts obtained for irv_height_desc at the various γ values have comparable FDR and higher TPR compared to Txp and Term. For the BrSimNorm dataset, barring the 0.01 FDR threshold, a similar trend is observed across the cuts compared to Term and Txp. It is important to note that since the reference units are different, the metrics are not directly comparable between the methods. We thus, next compared the number of transcripts belonging to the significant nodes in the lfc_desc cut that are also true positives but are not covered by either Txp or transcripts belonging to Term for the 0.1 FDR threshold. We find more than 2200 such transcripts while only 278 true positive transcripts exist in Term that are not covered by lfc_desc cut. These 2K transcripts map to around 828 inner nodes in the cut, which in total maps to more than 9500 transcripts. However, this cut allows us to recover signal w.r.t these true positive transcripts at a higher level in the tree which would have been lost by looking at just the transcripts.

**Table 3:** True Positive Rate and False Discovery Rate for the different methods at nominal FDR cutoffs 0.01, 0.05, 0.10 for the BrSimLow Dataset

Method	FDR			TPR		
	0.01	0.05	0.10	0.01	0.05	0.10
irv_height_desc(γ=0.05)	0.006	0.033	0.070	0.283	0.398	0.455
irv_height_desc(γ=0.10)	0.005	0.034	0.071	0.273	0.385	0.445
irv_height_desc(γ=0.50)	0.006	0.034	0.074	0.244	0.373	0.432
irv_height_desc(γ=1)	0.007	0.040	0.076	0.235	0.369	0.426
irv_height_desc(γ=5)	0.007	0.040	0.083	0.220	0.356	0.418
irv_height_desc(γ=10)	**0.001**	**0.024**	0.065	0.124	0.301	0.390
lfc_desc	0.006	0.037	0.081	**0.314**	**0.437**	**0.495**
Gene	0.002	0.025	**0.061**	0.256	0.413	0.491
Txp	0.008	0.039	0.080	0.208	0.345	0.407
Term	0.008	0.038	0.079	0.223	0.352	0.415

The cuts are obtained by optimizing the metric irv_height_desc at different γ values and lfc_desc on the Cons tree. The performance is also computed when the inferential units consist of genes, transcripts and terminus groups.

The entries in bold describe the best performing method across the different columns.

## Discussion

TreeTerminus organizes the transcripts in a tree structure, with the leaves representing the transcripts. The tree accounts for experiment-wide inferential uncertainty and provides the flexibility to analyze data at nodes that are at different levels of resolution that are best supported by data for the downstream analysis of interest. The cut given by the DP implementation on the objective functions used in this paper provides one possible way to get a set of nodes that can be used for downstream tasks. The DP is generalized and the base metric inside the objective function can be easily replaced by a user-defined function. Furthermore, the end user can also define a completely different approach to find a cut from the tree.

We have provided two different approaches for getting the transcript trees as output from an RNA-Seq experiment, Mean and Cons. They both cover a similar number of transcripts and have comparable performance across the different datasets for the various analyses that have been explored in this paper. They have superior performance compared to the consensus trees that were obtained when the constraints were kept on the various evaluation metrics and cover the largest number of transcripts. The sub-par performance for the other methods, especially ConsFiltES and Term perhaps indicate that these methods under-aggregate. While no other method exists that arrange the transcripts in a tree-like structure, for comparison we also provide the AC tree as a baseline, which is constructed using UPGMA—a well-known method for tree construction in phylogenetics. However, construction of the AC tree is both highly memory and time intensive, with the tree construction process not taking into account the equivalence class information. As a result, we observe that the nodes belonging to AC tree map to a large number of distinct genes and gene families, not offering much biological interpretation. The nodes belonging to trees constructed from TreeTerminus map to a smaller number of distinct genes and gene families, even though annotation of the underlying organism was not a part of the input.

Differential expression or differential abundance analysis is an important downstream application of RNA-Seq and other sequencing-based datasets. Various differential testing methods that leverage a tree structure^20^^,^^21^^,^^22^^,^^23^ have been proposed, reporting an increased power. In microbiome analysis, investigators have tried to associate nodes, located at different levels on the tree built through phylogenetic analysis, with a response variable of interest,^24^ with some using replicability as a metric to determine optimal levels of aggregation.^25^ We believe that the tree (unified) provided by TreeTerminus can be used with these methods for improved differential analysis in RNA-Seq. While we do perform differential testing on the cuts, they are not necessarily optimized for that task. Further, TreeTerminus in the future can also be extended to tagged-end scRNA-seq protocols where the trees will be constructed on genes rather than transcripts, owing to read mapping ambiguity at the gene level itself since the exons of one gene can overlap with exons or introns of other genes,^26^ as these are 3′- biased, often single end reads.

### Limitations of the study

There do exist some areas where the underlying tree construction methods can be improved further. Taking the mean of difference in inferential relative variance on all samples for constructing Mean tree might prevent transcripts from aggregation that had high uncertainty but were expressed only in the samples belonging to a population that was not in the majority in the experiment. This can be replaced by a weighted reduction in inferential relative variance, where the mean is computed over the samples in which the transcripts are expressed. Further, in the current implementation of TreeTerminus for the Mean tree, all samples’ inferential replicates are loaded in the memory, which might not scale well for an RNA-Seq experiment with large number of samples. A limitation with the Cons tree is the consensus tree algorithm that requires all the underlying input trees to span the same leaf set, causing us to modify the individual sample group trees that were provided as input. An alternative to consensus algorithms can be supertree methods, such as STELAR^27^ and FastRFS,^28^ which don’t require that all the input trees should cover the same leaf set. However, STELAR is very slow for trees that span large leaf sets and FastRFS provides an unrooted tree as an output, which makes using them as a direct replacement not trivial. Modifications to these approaches can be explored in the future.

## STAR★Methods

### Key resources table

**Table:** 

REAGENT or RESOURCE	SOURCE	IDENTIFIER
**Deposited data**		

Gencode V26, Transcript sequences fasta	Frankish et al.^29^	https://ftp.ebi.ac.uk/pub/databases/gencode/Gencode_human/release_26/gencode.v26.transcripts.fa.gz
Gencode M25, Transcript sequences fasta, reference genome fasta	Frankish et al.^29^	https://ftp.ebi.ac.uk/pub/databases/gencode/Gencode_mouse/release_M25
Pan_tro 3.0, ncRNA, cDNA, reference DNA	Cunningham et al.^30^	https://ftp.ensembl.org/pub/release-104/fasta/pan_troglodytes/
Mouse Muscle Dataset	Terry et al.^17^	https://www.ncbi.nlm.nih.gov/geo/query/acc.cgi?acc=GSE100505
Chimp Brain Dataset	Sousa et al.^18^	https://doi.org/10.7303/syn7067053

**Software and algorithms**		

Terminus v 0.1.0	Sarkar et al.^14^	https://github.com/COMBINE-lab/Terminus
Salmon v 1.5.2	Patro et al.^9^	https://github.com/COMBINE-lab/salmon
PySAT v 0.1.7.dev19	Ignatiev et al.^31^	https://github.com/pysathq/pysat
Snakemake v 6.6.1	Mölder et al.^32^	https://github.com/snakemake/snakemake
bedtools v 2.29.1	Quinlan and Hall^33^	https://github.com/arq5x/bedtools2
fastqc v 0.11.7	Andrews et al.^34^	https://github.com/s-andrews/FastQC
multiqc v 1.8.dev0	Ewels et al.^35^	https://github.com/ewels/MultiQC
R Packages	This study	https://doi.org/10.5281/zenodo.7807370
TreeTerminus	This study	https://github.com/COMBINE-lab/TreeTerminus/

### Resource availability

#### Lead contact

Further information and requests for resources should be directed to and will be fulfilled by the lead contact, Noor Pratap Singh (npsingh@umd.edu).

#### Materials availability

This study did not generate new unique reagents.

### Method details

We first briefly describe Terminus,^14^ as our method builds on top of it. The group step of Terminus takes as input an individual Salmon^9^ quantified sample from an RNA-Seq experiment and outputs a set of transcript groups. It makes use of the range-factorized equivalence classes ξ,^16^ and inferential replicates P obtained after running Salmon with the appropriate flags. An equivalence class denotes an association from a set of transcripts to a set of reads, that are mapped to all the transcripts in that set. A range-factorized equivalence class in addition also encodes the mapping quality, with a single class constituting a set of pairs $\left(t_{i},w_{i}\right)$ rather than just set of $t_{i}$, where $t_{i}$ denotes the transcript and $w_{i}$ represents the average conditional probability with which the fragments in the equivalence class arose from that transcript. The Terminus groups consist of transcripts that share large numbers of ambiguously-mapped fragments. It employs a union-find data structure, with the first step being to scan over ξ and group the transcripts that appear in the same set of equivalence classes and have near-identical conditional probability vectors using the Union operation. This ensures that these transcripts will belong to the same partition, when checked through the Find operation. It then constructs a graph with the transcripts as nodes, where an edge between any two nodes $v_{i},v_{j}$ implies that they co-occur in at least one equivalence class and have the edge score $s\left(v_{i},v_{j}\right)\leq \tau$ where

(Equation 1) $$s\left(v_{i},v_{j}\right)={\text{infRV}}_{i+j}-\frac{{\text{infRV}}_{i}+{\text{infRV}}_{j}}{2}$$

(Equation 2) $${\text{infRV}}_{i}=\frac{\max\left(\sigma_{p_{i}}^{2}-\mu_{p_{i}},0\right)}{\mu_{p_{i}}+pc}+d$$

$p_{i}$ are the posterior (Gibbs) replicates for transcript *i*, $\sigma_{p_{i}}^{2}$ and $\mu_{p_{i}}$ are the variance and mean over the posterior replicates, pc is the pseudocount (default is 5), *d* is a small global shift (default is 0.01) and τ is a threshold of difference in inferential relative variance. A min heap H is constructed over the edges keyed by $s\left(v_{i},v_{j}\right)$ and at each iteration *t* an edge with the lowest score is popped out till the iteration H becomes empty. If the partition(group) of any endpoint vertex for the popped edge has been modified but its corresponding score was not updated, then the edge is called stale. If the edge is not stale then the corresponding nodes $v_{i},v_{j}$ are grouped using the Union operation and the posterior samples for $v_{i}$ are updated with $p_{i}=p_{i}+p_{j}$, assuming i<j. The score $s\left(v_{i},x\right)$ is recomputed for all the vertices $\left\{x|x\in adj\left(v_{i}\right)\cup adj\left(v_{j}\right)-\left\{v_{i},v_{j}\right\}\right\}$ with $adj\left(v_{i}\right)$ denoting the neighbours of vertex *i* in the graph at iteration *t*. The edge with the updated $s\left(v_{i},x\right)$ is pushed to H, if $s\left(v_{i},x\right)\leq \tau$.

The consensus step outputs a set of transcript groups across samples using the groups obtained for the individual samples. The procedure involves creating a connected undirected graph on the transcripts, with an edge between any pair of transcripts denoting that they co-occur in a group in any sample and the edge weight denotes the count of the total number of samples in which the pair co-occurs in a group. To create the consensus groups, it finds the connected components on the updated graph obtained after only keeping the edges that occur in atleast a certain proportion of samples. This proportion is a user-defined parameter and by default has been set to 0.5.

#### TreeTerminus

The transcripts in a Terminus group consist of at least two transcripts that have reads multi-mapped to them, which is the source of uncertainty for transcript abundance estimation. TreeTerminus outputs a tree for each individual group, encoding the summarized order in which a set of transcripts should be aggregated within a group, such that across the samples, average uncertainty decreases as we ascend the tree created for that group.

TreeTerminus has a group step, that constructs transcript-trees for a single sample. It starts by creating a single node tree corresponding to each transcript. For any pair of transcripts/subgroups that are aggregated in the group step of Terminus, a binary tree is created with its children being the individual trees corresponding to the node-pairs that are grouped. The process of tree creation continues till the time heap H becomes empty. In addition, some of the constraints that Terminus imposed on transcripts/groups before they could be even considered for further grouping have also been relaxed. It is no longer necessary that a node $v_{i}$ should have $infRV\left(v_{i}\right)\geq \delta$ (called filtered or Filt) and that for a pair of nodes $v_{i},v_{j}$, $s\left(v_{i},v_{j}\right)\leq \tau$ (called early stop or ES). For the *N* groups that will be obtained from a sample *m*, a forest of *N* trees $T^{m}=\left\{T_{1m},\ldots ,T_{Nm}\right\}$ will be generated, such that for a group *g*, $\Lambda \left(g\right)=\Lambda \left(T_{gm}\right)$, with Λ representing the set of transcripts covered by a tree or a group and $\Delta^{m}$ represents all the trees belonging to sample *m*. We next propose and describe two different approaches to obtain trees, representative of all samples in the experiment.

##### Mean tree

This is a single step procedure where all the samples are processed together and a single tree is produced from all the samples w.r.t a group. The steps in the tree construction follow directly from what we have described above, with some modifications. To find transcripts that have near identical conditional probability weights across all the equivalence classes, the search space is expanded to all samples rather than a single sample. Later, when the graph across transcripts is constructed, an edge between any two nodes implies that they co-occur in the same equivalence class in at least one sample where the edge score $s^{\prime }\left(v_{i},v_{j}\right)$ is updated as, $s^{\prime }\left(v_{i},v_{j}\right)=\frac{\sum_{j=1}^{M}s_{j}\left(v_{i},v_{j}\right)}{M}$, and *M* represents the total number of samples in the experiment. The indicator function h(.) that determines whether a transcript is a good candidate for aggregation when considered in isolation is also updated as:

(Equation 3) $$h\left(v_{i}\right)=\left\{ \begin{array}{cc} 1 & \text{if }\overset{M}{\max_{j=1}}\;\mu_{ij}\geq 1\;\text{and}\;\max_{j=1}^{M}\;sp_{ij}\geq \lambda \\ 0 & \text{otherwise} \end{array}\right.$$

where $\mu_{ij}=\text{mean}\left(p_{ij}\right)$ and $sp_{ij}=\frac{\max\left(p_{ij}\right)-\min\left(p_{ij}\right)}{\text{mean}\left(p_{ij}\right)}$, with $p_{ij}$ denoting the posterior replicates for transcript *i* in sample *j* and λ is a user-defined parameter that is set to 0.1 that serves as a threshold for spread.

##### Consensus tree

Construction of consensus trees is more involved and takes as input the sample group trees obtained by running group step on each sample. Given a set of trees for a given transcript group across samples, we want to find a tree that summarizes the topological structure of the input trees. Such a tree is called consensus tree.^36^^,^^37^ There exists a wide variety of methods to get consensus trees, depending on the input and downstream applications.^37^^,^^38^^,^^39^^,^^40^ In this work we have used the majority rule extended or greedy consensus tree algorithm^38^^,^^41^ which we describe below.

Given a tree *T*, let V(T) denote the set of all nodes and Λ(T) denotes the set of transcripts covered by the tree *T*. For any node u∈V(T), T[u] is the subtree rooted at *u*, Λ(T[u]) is the cluster associated with it and C(T) represents the set of all clusters associated with tree *T*. Let *S* denote the collection of trees $\left(T_{1},T_{2},..T_{M}\right)$, with $\Lambda \left(T_{i}\right)=L,\forall i\in \left\{1,\ldots ,M\right\}$, aka all trees have the same leaf set. Let *X* be the set of all clusters that occur in *S* sorted by the decreasing order of the frequencies with which they occur in the trees. Construct a set *Y* of clusters as: Initialize Y=∅, then traverse *X* and for each cluster *C* encountered in this order, check if *C* and are pairwise compatible for all $C^{\prime }\in Y$, if yes then Y=Y∪{C}. A greedy or majority extended consensus tree of *S* is a tree *T* such that Λ(T)=L and C(T)=Y.

A constraint of a consensus tree algorithm is that it requires all the input trees should span the same leaf set. To obtain a consensus tree $T_{g}$ for group *g* in an experiment containing *M* samples, we want to provide group trees across samples $\Delta_{g}=\left\{T_{g1},\ldots ,T_{gM}\right\}$ as an input to the Majority Rule Extended Consensus Tree Algorithm, with $T_{gi}$ representing the tree for sample *i* for the group *g* and $\Lambda \left(g\right)=\Lambda \left(T_{gi}\right)$, ∀i∈{1,…,M}. Λ represents the transcripts covered by a tree or a group. However, a group covering a transcript set in one sample might not be preserved in other samples. We can have tree/s within a sample that covers a transcript set that overlaps but is not the same as the transcript(leaf) set in a tree from some other sample in the experiment. This is demonstrated in the example shown in Figure S1A. For the five different samples, we have trees that cover different transcript sets, covering overlapping transcripts. First sample contains the tree built on 5 transcripts. The second and fifth samples span the same set of 4 transcripts but are subsets of the transcripts covered by $T_{11}$. The third and fourth samples each have two trees containing transcripts that are also a subset of $\Lambda \left(T_{11}\right)$. Thus, we cannot directly apply the consensus tree algorithm on the trees obtained by the group step of TreeTerminus.

To resolve this issue, we thus first create a set of updated groups $G^{\prime }=\left\{g_{1}^{\prime },\ldots ,g_{N}^{\prime }\right\}$, with updated group $g_{i}^{\prime }$ representing a union of all transcripts for all trees across samples that contain overlapping transcripts. Further, the transcripts covered by any such tree should not contain any overlapping transcripts with the transcripts of any other updated group $g_{j}^{\prime }$ or: $\Lambda \left(g_{i}^{\prime }\right)=\overset{M}{\underset{m=1}{\cup }}\overset{N_{m}}{\underset{g=1}{\cup }}\Lambda \left(T_{gm}\right)$ where $\Lambda \left(T_{gm}\right)\subset \Lambda \left(g_{i}^{\prime }\right)$ and $\Lambda \left(T_{gm}\right)\cap \Lambda \left(g_{j}^{\prime }\right)=\emptyset$, ∀j∈{1,…,N},j≠i. To create the updated group $g_{i}^{\prime }$, we employ the union-find data structure. We scan through the trees across all samples and apply Union operation on the transcript set covered by the tree, which will group these transcripts covered. This ensures that the transcripts belonging to any two trees $T_{i},T_{j}$ across the samples where $\Lambda \left(T_{i}\right)\cap \Lambda \left(T_{j}\right)\neq \emptyset$ will be grouped together. For the toy example in Figure S1B, this leads to creation of the updated group spanning transcript set {1,2,3,4,5}. The consensus tree $T_{g_{i}^{\prime }}$ will be created for the updated group $g_{i}^{\prime }$, with $\Lambda \left(T_{g_{i}^{\prime }}\right)=\Lambda \left(g_{i}^{\prime }\right)$.

We next create trees $T_{g_{i}^{\prime }m}$ for every sample m∈{1,…M} w.r.t each updated group $g_{i}^{\prime }\in G^{\prime }$, such that tree $T_{g_{i}^{\prime }m}$ covers same set of transcripts as the updated group $g_{i}^{\prime }$ or $\Lambda \left(T_{g\prime_{i}m}\right)=\Lambda \left(g_{i}^{\prime }\right)$. Tree $T_{g\prime_{i}m}$ on the updated group $g\prime_{i}$ for sample *m* is created using the following steps:

1. For each sample *m*, all the trees $T_{gm}$ from $\Delta^{m}$ which cover transcripts that overlap with the transcripts covered by $g_{i}^{\prime }$ are extracted to form the set $\Delta_{g_{i}^{\prime }m}$ where $\{\Delta_{g_{i}^{\prime }m}=\left\{T_{1m},\ldots ,T_{dm}\right\}\subset \Delta^{m}$ | $\Lambda \left(T_{gm}\right)\subset \Lambda \left(g_{i}^{\prime }\right)$ ∀g∈{1,…,d} & $\Lambda \left(g_{i}^{\prime }\right)\cap \Lambda \left(T_{j}\right)=\emptyset$, m$\forall T_{j}\in \Delta^{m}-\Delta_{g_{i}^{\prime }}\}$.
2. A transcript set D is constructed that consists of transcripts covered by the updated group $g_{i}^{\prime }$ but not by the trees in $\Delta_{g_{i}^{\prime }m}$. Formally $D=\Lambda \left(g{,\prime }_{i}\right)-\overset{d}{\underset{i=1}{\cup }}\Lambda \left(T_{im}\right)$, with $T_{im}\in \Delta_{g_{im}^{'}}$.
3. If the set $\Delta_{g_{i}^{\prime }m}$ consists of only one tree $T_{gm}$ and D is empty, it implies that the tree $T_{gm}$ already covers the same transcript set as $g_{i}^{\prime }$ and thus we can safely output $T_{gm}$ as $T_{g_{im}^{'}}$.
4. Otherwise, an empty tree is constructed and added to it’s children are all the trees in $\Delta_{g_{i}^{\prime }m}$ along with the transcripts in the set D. This newly created tree forms $T_{g_{i}^{\prime }m}$.

For each group $g_{i}^{\prime }\in \left\{g_{1}^{\prime },\ldots ,g_{N}^{\prime }\right\}$, there now exists a set of *M* trees $\Delta_{g_{i}^{\prime }}=\left\{T_{g_{i}^{\prime }1},\ldots ,T_{g_{i}^{\prime }m}\right\}$, - one for every sample. PHYLIP’s^42^^,^^43^ implementation of Majority Rule Extended Consensus Algorithm is then applied on the set $\Delta_{g_{i}^{\prime }}$ to get the consensus tree $T_{g_{i}^{\prime }}$ for the updated group $g_{i}^{\prime }$. The motivation behind using the majority rule extended consensus algorithm is to provide a chance to preserve a topology present in set of samples that represent a phenotype not present in the majority of samples, which would have been ignored otherwise.

#### Creating a unified tree from the output of TreeTerminus

Given the output of TreeTerminus $\Delta =\left\{T_{g_{1}^{\prime }},\ldots ,T_{g_{N}^{\prime }}\right\}$, a unified tree $T_{U}$ is created such that its children consist of all the trees in Δ as shown in Figure S2. This is done through our R package beaveR, which also provides the option to append transcripts to the children of $T_{U}$ that are not covered by the trees in Δ but still should be considered for downstream analysis.

#### Solving objective functions to obtain discrete inferential units

We provide a dynamic programming (DP) approach that can be used to optimize different objective functions on a tree, following certain constraints. The DP solving a given objective function outputs a set of nodes in the tree *T* or cut $C=\left(c_{1},\ldots ,c_{d}\right)$, where *C* has the following properties:

• The union of leaf nodes belonging to nodes in the cut should cover all the leaf nodes in the tree *T* or U$\overset{d}{\underset{i=1}{\cup }}\Lambda \left(c_{i}\right)=\Lambda \left(T\right)$.
• The intersection of leaf nodes belonging to any two distinct nodes in the cuts should lead to an empty set or $\Lambda \left(c_{i}\right)\cap \Lambda \left(c_{j}\right)=\emptyset ,\forall i,j\in \left\{1\ldots d\right\}$.

The DP optimizes the objective functions of the following form to obtain the optimal value and a cut *C*:

$$\underset{C}{\mathrm{argmin}}f\left(C\right)\;\text{or}\;\underset{C}{\mathrm{argmax}}f\left(C\right)$$

$$\text{where}f\left(C\right)=\sum_{c\in C}\text{metric}\left(c\right)$$

where metric(c) represents the metric for node *c* in the tree *T*, whose sum we want to optimize over all nodes in the cut. We solve for f(C) using the procedure described in Algorithm 1. For a given metric of interest, it outputs the optimal value for the objective function and a set of nodes (cut), on which summing up the metric provides the optimal value. Algorithm 2 (CompOptVal) returns the optimal value for the objective function at a given node and Algorithm 3 (CompOptCut) returns the cut for a tree/subtree for that objective function.

Algorithm 1 **Finding optimal value of the objective function and the corresponding cut for a given metric**

**Input:**T,met_arr,type▷*T* is the tree, met_arr is the array containing the value of the metric for all nodes in the tree, type tells whether minimize or maximize the objective function

**Output:** $cut=\left[c_{1},\ldots ,c_{d}\right]$

**procedure** findOptValCut
  root←FindRoot(T) ▷ get the index corresponding to the root node in the tree
  t_nodes← length(*T*) ▷t_nodes are the total number of nodes in the tree
  cut←[]
  opt_arr←[0;t_nodes]
  **for** n in 0..t_nodes **do**
    opt_arr[n]← CompOptVal(T,n,met_arr,type) ▷. Optimal value at all nodes for the objective function
  end for
  CompOptCut(T,root,met_arr,opt_arr,cut).
  **return**cut
end procedure

Algorithm 2 **Function for finding optimal value of objective function at given node**

**Input:** T,node,met_arr,type

**Output:** opt_val_node

**procedure** compOptVal
  **if**node isLeaf **then**
    **return**met_arr[node]
  **end if**
    children←findChildren(T,node)
    child_opt←$\sum_{n\in children}$CompOptVal(n,met_arr,type)
  **if**type = min **then**
    **return**min(met_arr[node],child_opt)
  **else**
    **return**max(met_arr[node],child_opt)
  **end if**
**end procedure**

Algorithm 3 **Function for finding optimal cuts**

**Input:** T,node,opt_arr,met_arr,opt_cut

**Output:** $cut=\left[c_{1},\ldots ,c_{d}\right]$

**procedure** CompOptCut
  **if**opt_arr[node]=met_arr[node]**then**
    opt_cut.append (node)
    **return**opt_cut
  **end if**
  children←findChildren(T,node)
  **for**child in children **do**
    **return** CompOptCut(child,opt_arr,met_arr,opt_cut)
  **end for**
**end procedure**

We have solved for the objective functions employing two different metrics to obtain cuts on the tree $T_{U}$ which we describe in the section below.

##### Minimize mean infRV and height

- We minimize the sum for the node metric - sum of mean inferential relative variance and height weighted by γ; and then multiplied by the number of descendant leaf nodes. This metric is called irv_height_desc and is described formally below with the objective function as:

(Equation 4) $$\underset{C}{\mathrm{argmin}}\sum_{c\in C}\text{irv}\_\text{height}\_\text{desc}\left(c\right)$$

(Equation 5) irv_height_desc(c)=irv_height(c)∗|Λ(c)|

(Equation 6) irv_height(c)=mirv(c)+γheight(c)

mirv(c) is the mean inferential relative variance for a node across samples, height(*c*) is defined as the number of edges on the longest path from node *c* to its descendant leaf, Λ(c) represents the descendant leaves for a given node *c*, with |Λ(c)|=1, if *c* is a leaf node. The reason that the number of descendants of a node are multiplied is because the value of the objective function is dependent on the number of nodes in the cut and will be biased towards nodes with higher height as they end up replacing multiple lower height descendant nodes. To get the optimal value and desired cut for the above objective function we use the Procedure defined in Algorithm 1 as:

(Equation 7) $$\text{opt}\_\text{val},\text{cut}=FindOptValCut\left(T_{U},\text{irv}\_\text{height}\_\text{desc},\min\right)$$

##### Maximize log fold change

In this objective function, we maximize the sum of node metric - absolute log fold change using CPM (counts per million) multiplied by the number of the descendant leaves for that node. This metric is called lfc_desc and is described formally below with the objective function as:

(Equation 8) $$\underset{C}{\mathrm{argmax}}\sum_{c\in C}lfc\_desc\left(c\right)$$

(Equation 9) lfc_desc(c)=wlfc(c)·|Λ(c)|

where lfc is the $\log_{2}$ fold change. Assuming the samples can be grouped into two conditions represented by the sets $m_{1}$ and $m_{2}$, lfc is given by:

(Equation 10) $$lfc\left(c\right)=\text{median}\left\{\text{lfc}{\left(c\right)}_{k}\right\},k\in \left\{1..R\right\}$$

(Equation 11) $$\text{lfc}{\left(c\right)}_{k}=\log_{2}\left(\frac{\sum_{j=1}^{m_{1}}cpm{\left(c\right)}_{jk}}{\left|m_{1}\right|}+\text{pc}\right)-\log_{2}\left(\frac{\sum_{j=1}^{m_{2}}cpm{\left(c\right)}_{jk}}{\left|m_{2}\right|}+\text{pc}\right)$$

where *R* represents the total number of inferential replicates, $cpm{\left(c\right)}_{jk}$ represents *k*th inferential replicate’s CPM for node *c* of sample *j*. We again use the procedure defined in Algorithm 1 to get the optimal value and the cut as:

(Equation 12) $$\text{opt}\_\text{val},\text{cut}=FindOptValCut\left(T_{U},\text{wlfc}\_\text{desc},\max\right)$$

#### Datasets

To demonstrate the benefits of TreeTerminus, we ran it on both simulated and experimental datasets spanning different organisms.

#### Simulated human datasets

Polyester^44^ was used to generate simulated RNA-seq data. TPM estimates were extracted from GTEx V8 frontal cortex dataset with the distribution of mean and dispersion values derived from GEUVADIS samples.^45^ The process of read generation has been described in detail in.^46^ We generated 12 samples with 6 samples in each condition. All transcripts were differentially expressed for 10% of the genes with all having the same fold change (DGE) and genes had a single transcript differentially expressed (DTE). We created two variations of this simulation by varying the range of fold change - in the first variation we keep the same fold change as in^46^ and in the second variation, the maximum range of fold change is lowered from 6 to 3. The first variation is referred to as BrSimNorm and second is referred as BrSimLow.

#### Mouse muscle dataset

This dataset is taken from the skeletal muscle study GSE100505.^17^ In this paper, we have used 12 samples with 6 samples belonging to Atria and 6 to Tibialis Anterior, with the accession numbers provided in Table S1. All the samples belong to organism mus musculus. This dataset is referred as MouseMuscle.

#### Chimpanzee brain dataset

The final dataset that has been analyzed in our study is the RNA-Seq data from,^18^ SynapseID - syn7067053 collected from 5 Chimpanzees (Pan Troglodyte). We refer to this dataset as ChimpBrain. For each specimen, samples from 16 different tissues representing hippocampus, amygdala, cerebellar cortex, mediodorsal nucelus of thalamus, striatum and 11 areas of neocortex were sequenced. The samples belonging to the medial dorsal nucleus were removed before running TreeTerminus. The lfc change was computed by taking 5 cerebellum samples as the first group and remaining 68 samples belonging to other tissues as the second group. Batch effects were observed w.r.t specimen label and corrected using sva.^47^

#### Construction of anti-correlation tree

All transcripts that had 0 counts across the samples and 0 counts across the inferential replicates for any sample were removed. For each sample *k*, Pearson correlation $r_{ij}^{k}$ was computed between each pair of transcripts across the inferential replicates. $r_{ij}^{k}$ is multiplied by −1 to give $r_{ij}^{k\prime }$ so that any pair of transcripts that had the highest negative correlation now have the largest positive correlation. To convert (anti)correlation into distance, $r_{ij}^{k\prime }$ is transformed as $d_{ij}^{k}=\left(1-r_{ij}^{k\prime }\right)/2$ or $d_{ij}^{k}=\frac{1+r_{ij}^{k}}{2}$. Unweighted Pair Group Method with Arithmetic Mean (UPGMA)^19^ was used to create the anti-correlation tree with the implementation derived from the R package phangorn.^48^ The mean for $d_{ij}^{k}$ across samples is computed and fed as input to UPGMA in order to get the final tree. The AC tree could also have been created for every sample and then provided as input to the consensus tree algorithm to get the final tree, however consensus tree algorithms do not scale well on the number of leaves. Since for most cases the number of leaves(transcripts) on trees would be in the order $10^{4}-10^{5}$, consensus tree algorithms would have taken a lot of time to converge.

#### Mapping to gene families

The gene family labels for a gene were extracted using R package biomaRt^49^ using Ensembl version 101. We want to extract the total number of unique gene families to which an inner node maps. A gene can map to more than one gene family and when an inner node maps to multiple genes; with atleast one gene mapping to more than one family, finding the unique number of gene families for that node is not trivial. Simply, taking a union of gene families across genes for an inner node is undesirable since this might lead to a situation where number of reported gene families are larger than the number of mapped genes for some nodes. Between different trees, the interpretation of the distribution of the number of gene families to which an inner node maps will also be biased towards the tree that has more nodes containing genes mapping to multiple gene families. Thus, in order to find the number of unique gene families associated with a node, we formulate this as the minimial hitting set problem.^50^ We want to find the minimum number of gene families for a node in the tree, whose intersection with the gene families associated with every gene for that node leads to a non-empty set. We use the implementation provided by the Python library PySAT^31^ to solve the minimal hitting set problem.

### Quantification and statistical analysis

For the Chimpanzee dataset, only bam files were available as the raw data which were converted into fastq using bamToFastq in bedtools.^33^ The quality control analysis for Chimpanzee and mice dataset was done using fastqc^34^ and multiqc.^35^ For creating the salmon indexes, gencode versions v26, M25 and Pan_tro 3.0 were used for human, mice and chimpanzee datasets. Salmon was used for quantification and generating 100 Gibbs replicates for each sample with a thinning factor of 100. All the pipelines used for analysis in this paper were created using Snakemake.^51^

#### Differential expression analysis

Differential expression analysis is carried out on both the simulated datasets using Swish.^15^ It is performed individually for the cuts obtained on the Cons tree by optimizing the objective functions defined in the section above. The inner nodes belonging to a cut are directly used as the inferential units for the analysis, and their underlying descendant transcripts are not considered. The performance is evaluated using True Positive and False Discovery rates computed at different nominal FDR thresholds (0.01, 0.05, 0.1). The performance is also evaluated when the inferential units consist of genes, transcripts(Txp), and Terminus groups(Term). The true differential status of a transcript group is determined by computing lfc on its aggregated transcript counts w.r.t each condition in the sim.counts.mat matrix that is created during the generating the input for Polyester^44^ and checking if |lfc| is beyond a threshold. It is important to keep in mind that these evaluation metrics are not directly comparable across the methods as the base reference units are different.

## Data Availability

• The paper analyzes simulated and existing publicly available data. The script to generate the simulated data has been uploaded to Zenodo with the DOI listed in the key resources table. The study accession ids for the experimental datasets are also listed in the key resources table. To obtain the raw Chimpanzee brain datasets, access has to be requested from the PsychEncode Portal. • TreeTerminus is publicly available online from https://github.com/COMBINE-lab/TreeTerminus. • The R Package beaveR is publicly available online from https://github.com/NPSDC/beaveR.
