# Supplementary material for: TreeTerminus —creating transcript trees using inferential replicate counts
Source: iScience. 2023 May 25;26(6):106961. doi: 10.1016/j.isci.2023.106961 (PMC10291472; doi:10.1016/j.isci.2023.106961)
Supplement: Document S1. Figures S1–S10 and Tables S1–S8 [file mmc1.pdf]

iScience, Volume *26*

Supplemental information

TreeTerminus —creating transcript trees using inferential replicate counts

Noor Pratap Singh, Michael I. Love, and Rob Patro

S1. Tables

Table S1: **Accession IDs for the tissue samples for the `MouseMuscle` dataset that have been used for analysis in this paper**, related to STAR Methods.

**Accession ID**	**TissueName**
GEO:SRR5758624	Atria
GEO:SRR5758625	Atria
GEO:SRR5758626	Atria
GEO:SRR5758627	Atria
GEO:SRR5758628	Atria
GEO:SRR5758629	Atria
GEO:SRR5758702	TA
GEO:SRR5758703	TA
GEO:SRR5758704	TA
GEO:SRR5758705	TA
GEO:SRR5758706	TA
GEO:SRR5758707	TA

Table S2: **Peak memory usage and running time for the different steps of Terminus and TreeTerminus (both `Mean` and `Cons` modes) on `MouseMuscle` dataset**, related to Table 1.

**Method**	**Terminus**		**TreeTerminus (Mean)**	**TreeTerminus (Cons)**	
	Group	Consensus	Group	Group	Consensus
Peak Memory (MB)	163	1950	2343	337	278
Time (h:m:s)	0:12:58	0:01:40	0:02:18	0:11:34	0:21:49

Table S3: **Median of mean inferential variance (MIRV) of the inner nodes for different trees stratified by their height for the `BrSimNorm` dataset.** All nodes with height larger than 5 have been labelled as 5, related to Figure 2.

**Tree**	**2**	**3**	**4**	**5**
Mean	0.17	0.11	0.09	0.07
Cons	0.19	0.12	0.10	0.07
AC	0.52	0.32	0.21	0.12
ConsFilt	0.44	0.35	0.19	0.10
ConsFiltES	0.89	0.56	0.42	0.26

Table S4: **Median of mean inferential variance (MIRV) of the inner nodes for different trees stratified by their height for the `BrSimLow` dataset.** All nodes with a height larger than 5 have been labelled as 5, related to Figure 2.

**Tree**	**2**	**3**	**4**	**5**
Mean	0.17	0.11	0.09	0.07
Cons	0.19	0.12	0.10	0.07
AC	0.52	0.33	0.21	0.13
ConsFilt	0.44	0.35	0.19	0.10
ConsFiltES	0.90	0.57	0.42	0.30

Table S5: **Median of mean inferential variance (MIRV) of the inner nodes for different trees stratified by their height for the `MouseMuscle` dataset.** All nodes with a height larger than 5 have been labelled as 5, related to Figure 2.

**Tree**	**2**	**3**	**4**	**5**
Mean	0.12	0.09	0.08	0.07
Cons	0.12	0.09	0.08	0.07
AC	0.47	0.35	0.24	0.19
ConsFilt	0.24	0.14	0.10	0.08
ConsFiltES	0.68	0.54	0.29	0.02

Table S6: **Median of mean inferential variance (MIRV) of the inner nodes for different trees stratified by their height for the `ChimpBrain` dataset.** All nodes with a height larger than 5 have been labelled as 5, related to Figure 2.

**Tree**	**2**	**3**	**4**	**5**
Mean	0.07	0.07	0.07	0.07
Cons	0.08	0.07	0.07	0.07
AC	0.09	0.07	0.06	0.06
ConsFilt	0.09	0.07	0.07	0.07
ConsFiltES	0.37	0.17	0.12	0.10

Table S7: **Total number of inner nodes mapping to more than** 100 **genes for different datasets**, related to Figure 3.

**Tree**	**BrSimNorm**	**BrSimLow**	**MouseMuscle**	**ChimpBrain**
Mean	0	0	113	0
Cons	0	0	44	0
AC	717	719	696	304
ConsFilt	0	0	37	0
ConsFiltES	0	0	36	0

Table S8: **True Positive Rate and False Discovery Rate for the different methods at nominal FDR cutoffs 0.01, 0.05, 0.10 for the `BrSimNorm` Dataset.** The cuts are obtained by optimizing the metric `irv_height_desc` at different γ values and `lfc_desc` on the `Cons` tree. The performance is also computed when the inferential units consist of genes, transcripts and terminus groups, related to Table 2.

**Method**	FDR			TPR		
	0.01	0.05	0.10	0.01	0.05	0.10
`irv_height_desc`($\gamma = 0.05$)	0.011	0.042	0.084	0.629	0.731	0.775
`irv_height_desc`($\gamma = 0.10$)	0.010	0.037	0.075	0.624	0.728	0.772
`irv_height_desc`($\gamma = 1$)	0.002	0.035	0.077	0.392	0.718	0.778
`irv_height_desc`($\gamma = 5$)	0.002	0.037	0.080	0.360	0.709	0.771
`irv_height_desc`($\gamma = 10$)	0.010	0.044	0.081	0.593	0.712	0.766
`lfc_desc`	**0.001**	0.038	0.080	0.501	**0.744**	**0.796**
`Gene`	0.009	**0.031**	**0.058**	**0.627**	0.706	0.739
`Txp`	0.010	0.037	0.074	0.579	0.695	0.751
`Term`	0.009	0.038	0.076	0.593	0.712	0.766

S2. Figures

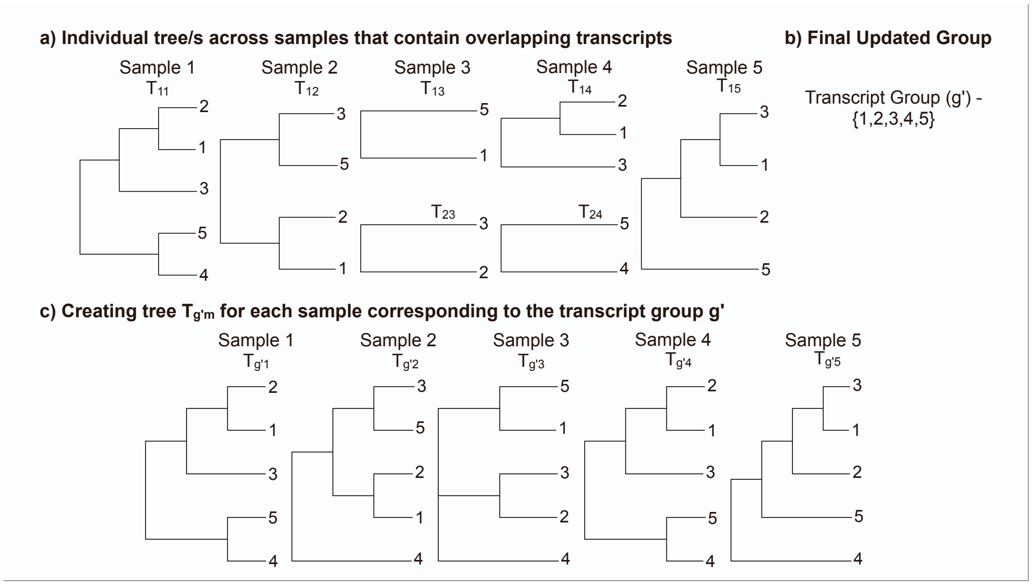

Figure S1: **Example of a sample group to demonstrate why consensus step cannot be directly applied on the trees obtained from group step for samples and the modifications made to apply the consensus tree algorithm**. **a** Trees across the 5 different samples that contain overlapping transcripts. Each tree is labelled as T_{gm}, where m denotes the sample and g denotes a group in that sample. **b** Updated group that is a superset of all the transcripts in the individual trees across samples. **c** Tree created for each sample w.r.t updated group g', with $\Lambda(T_g') = \Lambda(g')$, related to STAR Methods.

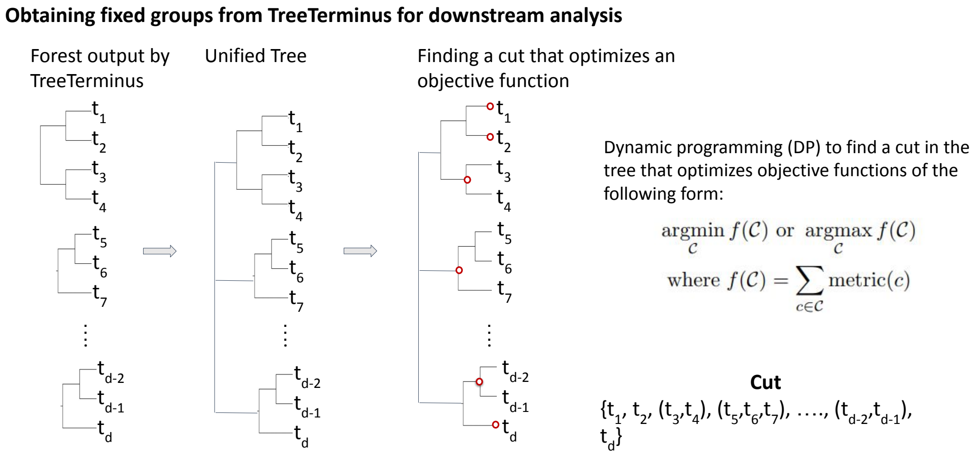

Figure S2: **Demonstration of creating a unified tree from the forest obtained as the output of TreeTerminus**. The unified tree can then be used for optimizing an objective function of interest in order to obtain a cut for downstream analysis, related to STAR Methods.

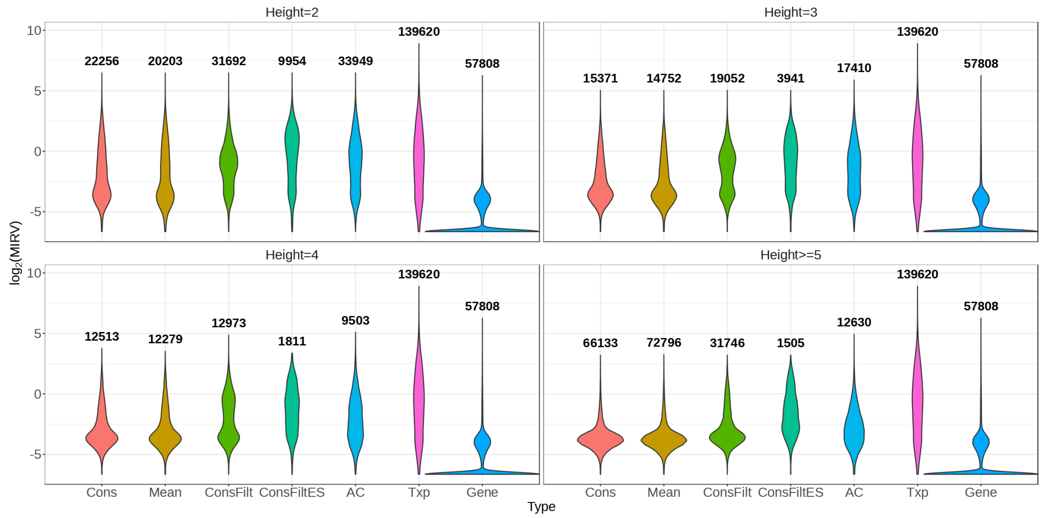

Figure S3: **Distribution of $\log_2$ MIRV (mean inferential variance) across samples for the inner nodes stratified by their height for different trees for the `BrSimLow` Dataset**. The total number of inner nodes belonging to a method at a given height is written on top of the violin plot. Also plotted for comparison at each height is the distribution of MIRV for the transcripts and genes, related to Figure 2.

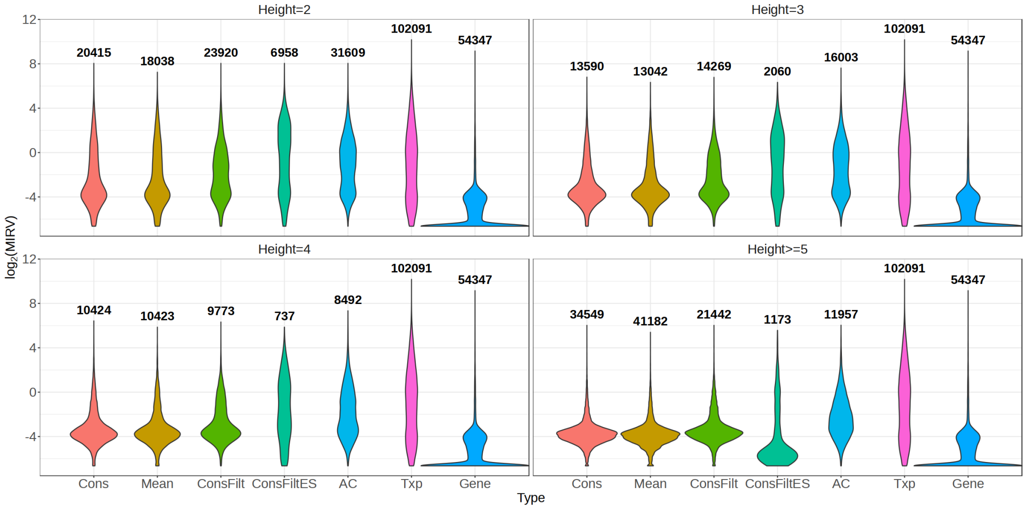

Figure S4: **Distribution of $\log_2$ MIRV (mean inferential variance) across the inner nodes stratified by their height for different trees for the** `MouseMuscle` **dataset**. The total number of inner nodes belonging to a method at a given height is written on top of the violin plot. Also plotted for comparison at each height is the distribution of lg of MIRV for the transcripts and genes, related to Figure 2.

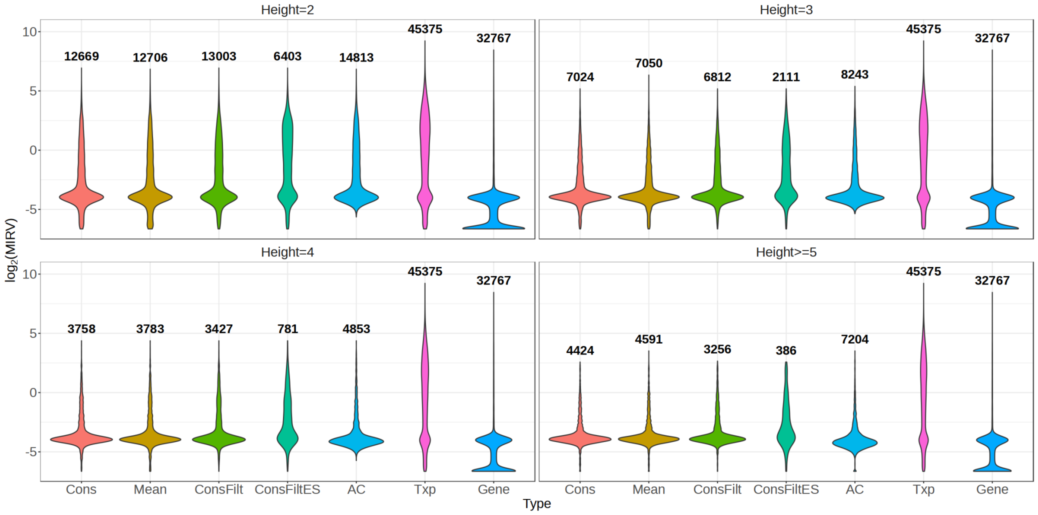

Figure S5: **Distribution of $\log_2$ MIRV (mean inferential variance) across samples for the inner nodes stratified by their height for different trees for the** `ChimpBrain` **dataset**. The total number of inner nodes belonging to a method at a given height is written on top of the violin plot. Also plotted for comparison at each height is the distribution of MIRV for the transcripts and genes, related to Figure 2.

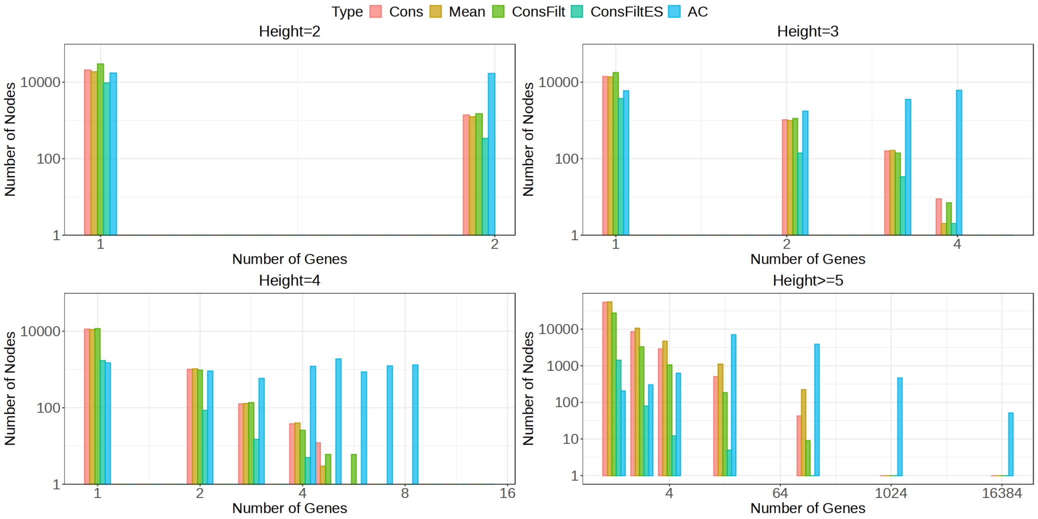

Figure S6: **Comparison of different tree methods with respect to the number of genes to which an inner node in the tree maps for the `BrSimLow` dataset stratified by their height**. The x-axis represents the number of unique genes that transcripts belonging to the inner nodes map to and the y-axis represents the frequency of such mappings at a given height for a tree. For all the inner nodes located at a height greater than or equal to 5, the number of unique genes was binned using the set $\{1, 2, 4, 16, 128, 1024, 16384\}$, with the bin representing the number of unique genes less than or equal to the bin but larger than the bin left to it, related to Figure 3.

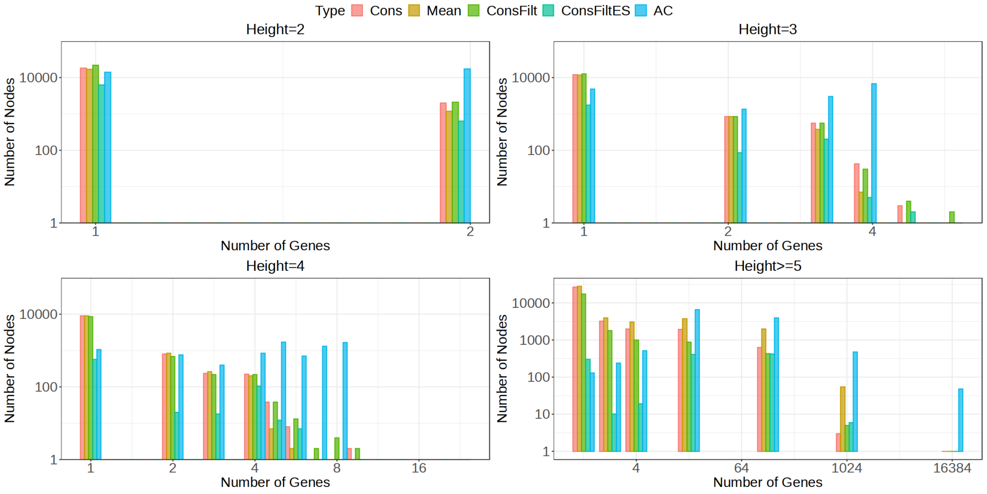

Figure S7: **Comparison of different tree methods with respect to the number of genes to which an inner node in the tree maps for the `MouseMuscle` dataset stratified by their height**. The x-axis represents the number of unique genes that transcripts belonging to the inner nodes map to and the y-axis represents the frequency of such mappings at a given height for a tree. For all the inner nodes located at a height greater than or equal to 5, the number of unique genes was binned using the set $\{1, 2, 4, 16, 128, 1024, 16384\}$, with the bin representing the number of unique genes less than or equal to the bin but larger than the bin left to it, related to Figure 3.

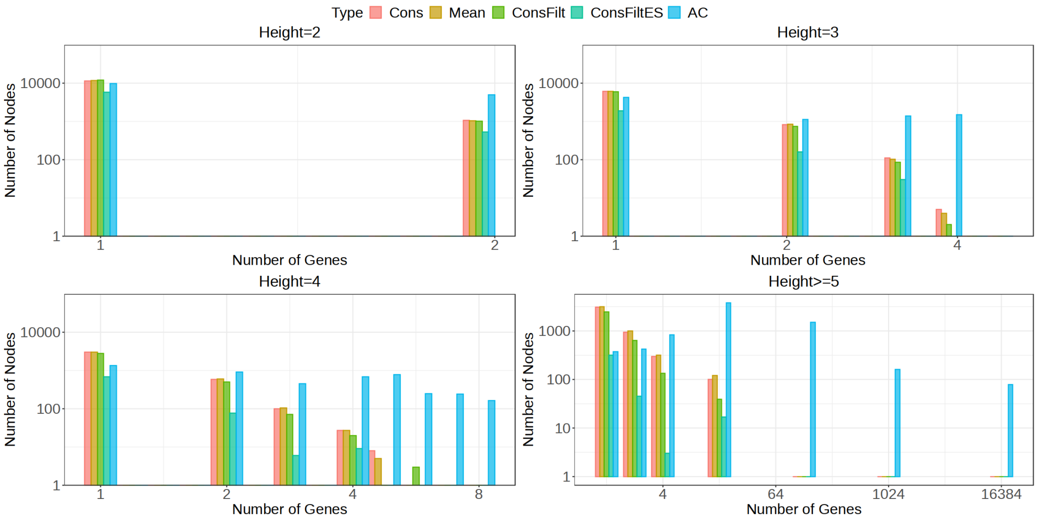

Figure S8: **Comparison of different tree methods with respect to the number of genes to which an inner node in the tree maps for the `ChimpBrain` dataset stratified by their height**. The x-axis represents the number of unique genes that transcripts belonging to the inner nodes map to and the y-axis represents the frequency of such mappings at a given height for a tree. For all the inner nodes located at a height greater than or equal to 5, the number of unique genes was binned using the set $\{1, 2, 4, 16, 128, 1024, 16384\}$, with the bin representing the number of unique genes less than or equal to the bin but larger than the bin left to it, related to Figure 3.

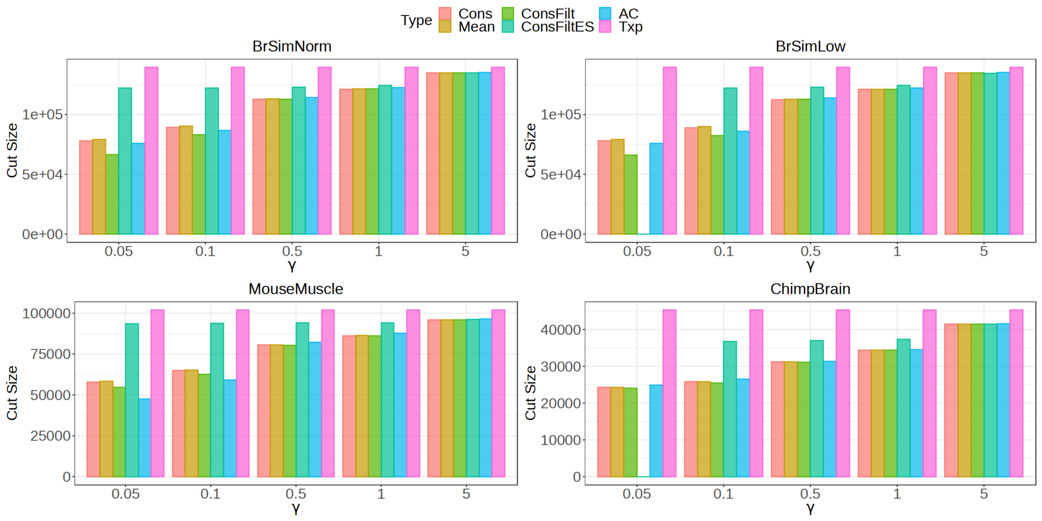

Figure S9: **Distribution of the size of cuts on the different datasets obtained after solving for the objective function that minimizes the sum of metric(`irv_height_desc`) for the nodes in a cut**. For each method, the distribution is plotted for a range of γ values. Also plotted for comparison are the total number of transcripts/leaves `Txp`, related to Figure 5.

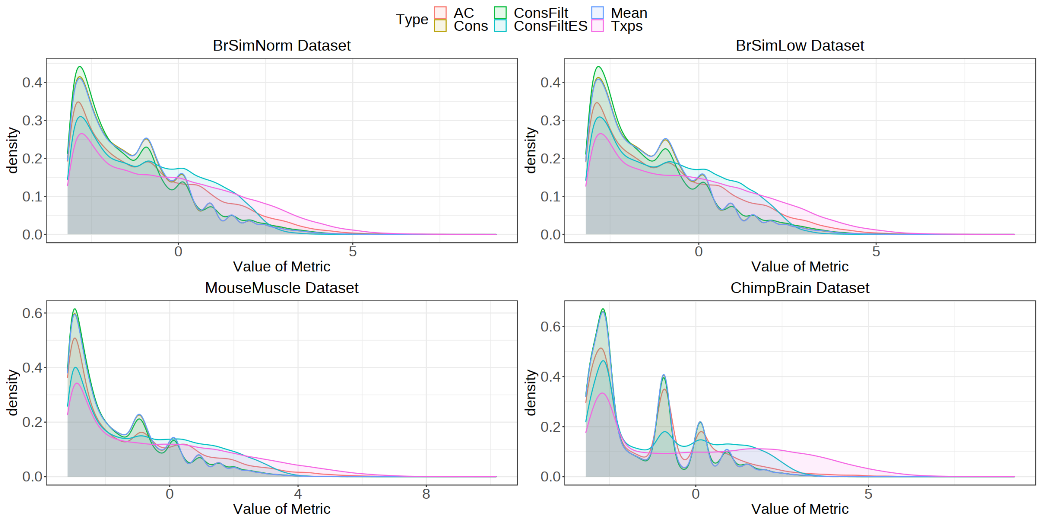

Figure S10: **Distribution of the** lg **of the metric(`irv_height_desc`) for the nodes in the cut obtained after minimizing for the objective function using `irv_height_desc` as the underlying metric across trees on the different datasets**. The metric has been computed using $\gamma = 0.1$. Also plotted for comparison is the distribution of `irv_height_desc` for transcripts, related to Figure 6.
